# Clinical and laboratory predictive markers for acute dengue infection

**DOI:** 10.1186/1423-0127-20-75

**Published:** 2013-10-20

**Authors:** Tzong-Shiann Ho, Shih-Min Wang, Yee-Shin Lin, Ching-Chuan Liu

**Affiliations:** 1 Institute of Clinical Medicine, College of Medicine, National Cheng Kung University, Tainan, Taiwan; 2 Department of Emergency Medicine, National Cheng Kung University Hospital, College of Medicine, National Cheng Kung University, Tainan, Taiwan; 3 Department of Pediatrics, National Cheng Kung University Hospital, College of Medicine, National Cheng Kung University, Tainan, Taiwan; 4 Department of Microbiology and Immunology, College of Medicine, National Cheng Kung University, Tainan, Taiwan; 5 Center of Infectious Diseases and Signaling Research, National Cheng Kung University, Tainan, Taiwan

**Keywords:** Dengue, Early diagnosis, Predictive markers

## Abstract

**Background:**

Early diagnosis of dengue virus infection during the febrile stage is essential for adjusting appropriate management. This study is to identify the predictive markers of clinical and laboratory findings in the acute stage of dengue infection during a major outbreak of dengue virus type 1 that occurred in southern Taiwan during 2007. A retrospective, hospital-based study was conducted at a university hospital in southern Taiwan from January to December, 2007. Patient who was reported for clinically suspected dengue infection was enrolled. Laboratory-positive dengue cases are confirmed by enzyme-linked immunosorbent assay of specific dengue IgM, fourfold increase of dengue-specific IgG titers in convalescent serum, or by reverse transcription-polymerase chain reaction (RT-PCR) of dengue virus.

**Results:**

The suspected dengue cases consist of 100 children (≤ 18 years) and 481 adults. Among the 581 patients, 67 (67%) children and 309 (64.2%) adults were laboratory-confirmed. Patients who had laboratory indeterminate were excluded. Most cases were uncomplicated and 3.8% of children and 2.9% of adults developed dengue hemorrhagic fever or dengue shock syndrome (DHF/DSS). The overall mortality rate in those with DHF/DSS was 7.1%, and the average duration of hospitalization was 20 days. The most common symptoms/signs at admission were myalgia (46.8%), petechiae (36.9%) and nausea/vomiting (33.5%). The most notable laboratory findings included leukopenia (2966 ± 1896/cmm), thrombocytopenia (102 ± 45 × 10^3^/cmm), prolonged activated partial thromboplastin time (aPTT) (45 ± 10 s), and elevated serum levels of aminotransferase (AST, 166 ± 208 U/L; ALT, 82 ± 103 U/L) and low C - reactive protein (CRP) (6 ± 11 mg/L). Based on the clinical features for predicting laboratory-confirmed dengue infection, the sensitivities of typical rash, myalgia, and positive tourniquet test are 59.2%, 46.8%, and 34.2%, while the specificities for above features are 75.4%, 53.5% and 100%, respectively. The positive predictive value (PPV) for combination of leukopenia, thrombocytopenia (< 150 × 10^3^/cmm), elevated aminotransferase (AST/ALT > 1.5) and low CRP (< 20 mg/L) is 89.5%, while the negative predictive value is 37.4%. Furthermore, the PPV of the combination was increased to 93.1% by adding prolonged aPTT (>38 secs).

**Conclusions:**

Leukopenia, thrombocytopenia, elevated aminotransferases, low CRP and prolonged aPTT, were useful predictive markers for early diagnosis of dengue infection during a large outbreak in southern Taiwan.

## Background

Dengue disease is an acute infectious disease caused by four serotypes of dengue virus, and is the most prevalent mosquito-borne viral disease in humans, occurring in tropical and subtropical countries of the world where over 2.5 billion people are at risk of infection [1]. The World Health Organization has estimated 50 million cases of dengue fever and several hundred thousand cases of dengue hemorrhagic fever occur each year, depending on the epidemic activity [2]. Some 1.8 billion of the population at risk for dengue worldwide live in member states of the WHO South-East Asia Region and Western Pacific Region, which bear nearly 75% of the current global disease burden due to dengue [3].

Dengue has a wide spectrum of clinical presentations, often with unpredictable clinical evolution and outcome. While most patients recover following a self-limiting non-severe clinical course, a small proportion progress to severe disease, mostly characterized by plasma leakage with or without hemorrhage. Early recognition of dengue is challenging because the initial symptoms are often non-specific, viremia may be below detectable levels and serological tests confirm dengue late in the course of illness [4]. Prompt diagnosis during the febrile stage is essential for adjusting appropriate management [5].

In endemic areas such as Southeast Asia or Latin America, dengue hemorrhagic fever is the leading cause of hospitalization and death among children with secondary infection. In different areas with a recent introduction of the virus or with no endemicity, the age distribution of dengue hemorrhagic fever cases is different with an increasing number of adults with DHF [6]. In 1987, a major dengue outbreak occurred in southern Taiwan [7]. Several major dengue endemics with various clinical characteristics and serotypes were observed in Taiwan during the past two decades [8,9].

Dengue is a category 2 notified infectious disease in Taiwan; the physicians are obliged to report the suspected dengue cases to the local health department within 24 hours of clinical diagnosis. Contacts of confirmed cases are also obliged to test their blood for dengue virus infection. Reliably identifying dengue patients early in their clinical course could help direct patient management and reduce the transmission of dengue virus in a community. Timely identification of dengue infection would enable healthcare providers potentially to prevent additional cases among close contacts by urging patients with a positive dengue screening test to use personal protection measures against mosquito bites [4]. However, there are no accepted clinical guidelines for the recognition of early-stage dengue infection. There is also no consensus as to whether clinical features can be used to distinguish dengue infection from other febrile illness [10-14].

This study is aimed to identify the predictive markers of clinical and laboratory findings in the early stage of dengue infection during the outbreak in Southern Taiwan in 2007.

## Methods

### Case definition

A retrospective, hospital-based study was conducted at National Cheng Kung University Hospital from Jan. to Dec., 2007. Patient who was reported for clinically suspected dengue infection was enrolled. Laboratory-positive dengue cases are confirmed by enzyme-linked immunosorbent assay of specific dengue IgM, fourfold increase of dengue-specific IgG titers in convalescent serum, or by reverse transcription-polymerase chain reaction (RT-PCR) of dengue virus. Pediatric patients are defined as patients who younger than 18 years.

**Figure 1:**
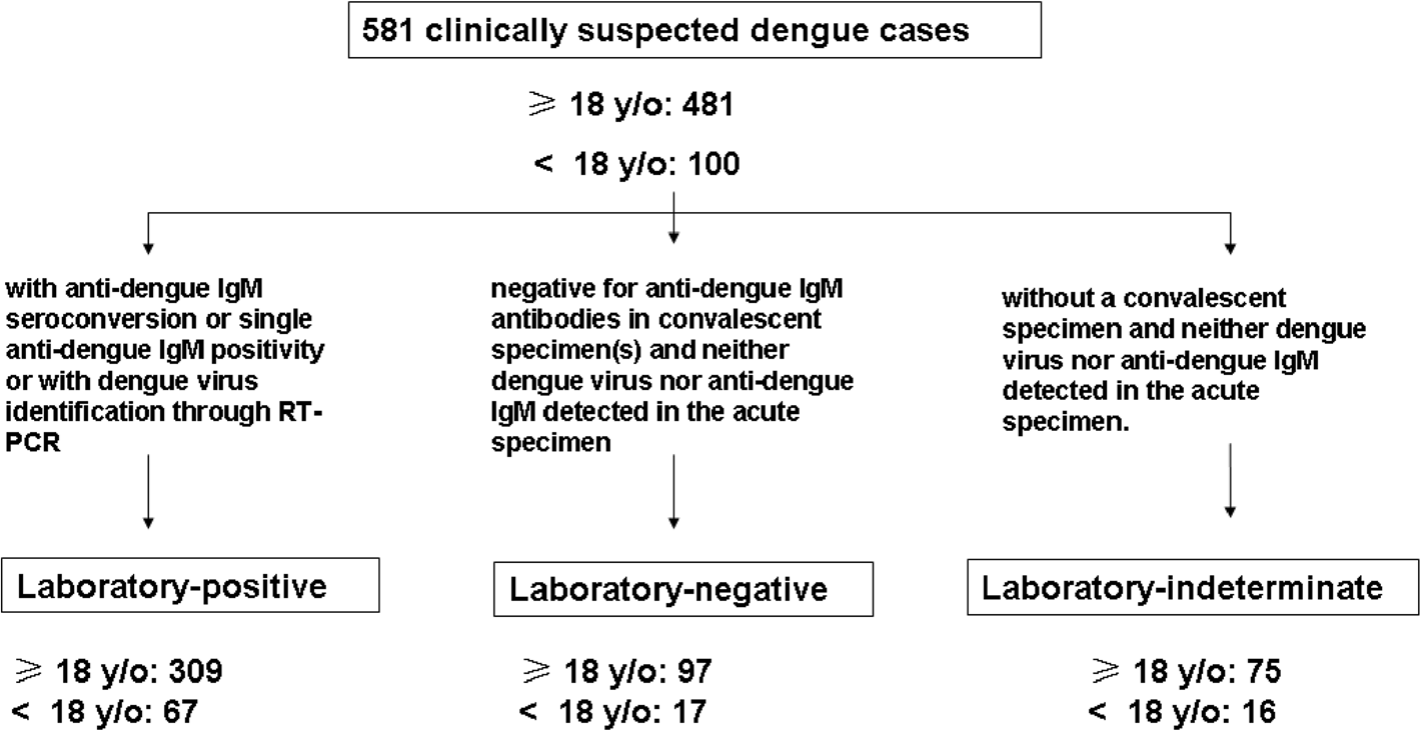
Category algorithm for 581 clinically suspected dengue cases during the 2007 outbreak in southern Taiwan.

Suspected dengue cases were defined as: patients with reported or documented fever of ≥38°C of less than 7 days duration and two or more symptoms or signs (headache, rash, eye pain, myalgia, arthralgia, hypotension, hemorrhage, hemoconcentration (elevated hematocrit ≥20% for age and gender, or equivalent drop in hematocrit (≥20% from baseline) after volume replacement therapy), thrombocytopenia (platelet count <150 × 10^3^/cmm) or for whom a physician suspected dengue for any reason. All clinically suspected dengue cases were categorized into three following groups (Figure 1): (a) laboratory-positive dengue case: suspected dengue case with anti-dengue IgM seroconversion or single anti-dengue IgM positivity or with dengue virus identification through RT-PCR. (b) laboratory-negative dengue case: suspected dengue negative for anti-dengue IgM antibodies in convalescent specimen; neither dengue virus nor anti-dengue IgM detected in the acute specimen. (c) laboratory-indeterminate dengue case: suspected dengue case without a convalescent specimen; neither dengue virus nor anti-dengue IgM detected in the acute specimen. Data from medical records were also collected, including demographic information, data on the timing of hospital visits, comorbidities, clinical features, and laboratory data. Clinical symptoms/signs such as myalgia, petechiae, skin rashes, headache, itching skin, nausea/vomiting, diarrhea were recorded. Positive tourniquet test [10] was defined if there are more than 20 petechiae in a defined 2.5-cm^2^ area. Cut-off values for laboratory tests were defined as followings: leukopenia (white blood cell count < 4000/cmm), thrombocytopenia (platelet count < 150 × 10^3^/cmm) prolonged activated partial thromboplastin time (aPTT) (> 38 sec), elevated serum aminotransferase levels (aspartate aminotransferase (AST) or alanine aminotransferase (ALT) >39 U/L) and low C-reactive protein (CRP) (< 20 mg/L). The chart review followed published guidelines on retrospective chart review methods to ensure accurate data abstraction and to limit the biases inherent to such studies [15]. The institutional review board of National Cheng Kung University Hospital approved the study protocol.

### Sample collection and dengue diagnosis

The collection of serum samples and laboratory diagnosis are performed for public health surveillance purposes and enforced by the Communicable Disease Prevention Act. A modified E/M-specific captured IgM and IgG ELISA was performed to measure the dengue virus (DENV) and Japanese encephalitis virus (JEV)-specific IgM and IgG antibodies as previously described [16-18]. The assay was performed by (1) measurement of DENV- and JEV-specific IgM and IgG antibodies of each serum sample simultaneously in the same ELISA plates, (2) selection of a monoclonal antibody having similar affinity to both JEV and the four DENV serotypes, (3) addition of virus-infected cell culture supernatants containing equal concentration of DENV or JEV in each well, and (4) captured IgM or IgG antibodies incubated to a cocktail of mixed viral antigens and the monoclonal antibody in a single step. A one-step SYBR Green I real-time RT-PCR (QuantiTect SYBR Green RT-PCR kit; Qiagen, Hilden, Germany) was performed in the Mx4000TM quantitative PCR system (Stratagene, La Jolla, CA) to detect and differentiate dengue virus serotypes in acute-phase serum samples, as described previously [17,18].

### Statistical analysis

Statistical analyses were performed using Fisher’s exact test for categorical variables and the t test for continuous variables. Clinical and laboratory findings were compared using SPSS version 11.5 (SPSS, Chicago, IL, USA). p values less than 0.05 were considered significant. The sensitivity and specificity of the items for predicting dengue infection were determined for each assigned cut-off value.

## Results

Dengue virus type 1 (DENV-1) was most common serotype detected by RT-PCR in this study (93% in adults, 96% in children), whereas DENV-2 was also detected. Most cases were uncomplicated and only 3.8% of children and 2.9% of adults developed dengue hemorrhagic fever or dengue shock syndrome (DHF/DSS). The overall yield rate for RT-PCR was 49.2% (185/376). The positive rate of RT-PCR at admission was similar between adult and pediatric groups (51% vs. 40%, p = 0.317). Most of the patient was hospitalized (79% in adults vs. 78% in children, p = 0.655) for an average of 3.4 ~ 4.1 days (4.1 ± 3.1 days in adults vs. 3.4 ± 2.6 days in children, p = 0.204) (Table 1). The overall mortality rate in those with DHF/DSS was 7.1%, and the mean duration of hospitalization was 20 days.

**Table 1:** Demographic characteristics and diagnosis of 376 dengue patients during the 2007 outbreak in southern Taiwan

	**≥ 18 y/o**	**<18 y/o**	P*
**Total (n,%)**	309 (82.1%)	67(17.9%)	
**Gender**			
**Male**	145 (46.9%)	45 (64.3%)	0.088
**Female**	164 (53.1%)	22 (35.7%)	
Age (years)**	48 (19–83)	14 (2–18)	
**Hospitalization (%)**	79%	78%	0.655
**Hospital stay (days)**	4.1 ± 3.1	3.4 ± 2.6	0.204
**Diagnosis**			
**Dengue Fever**	297 (96.2%)	65 (97.1%)	
**DHF/DSS**	12 (3.8%)	2 (2.9%)	

DHF/DSS: dengue hemorrhagic fever/dengue shock syndrome.

*Chi-square test or t-test, **median (range).

**Figure 2:**
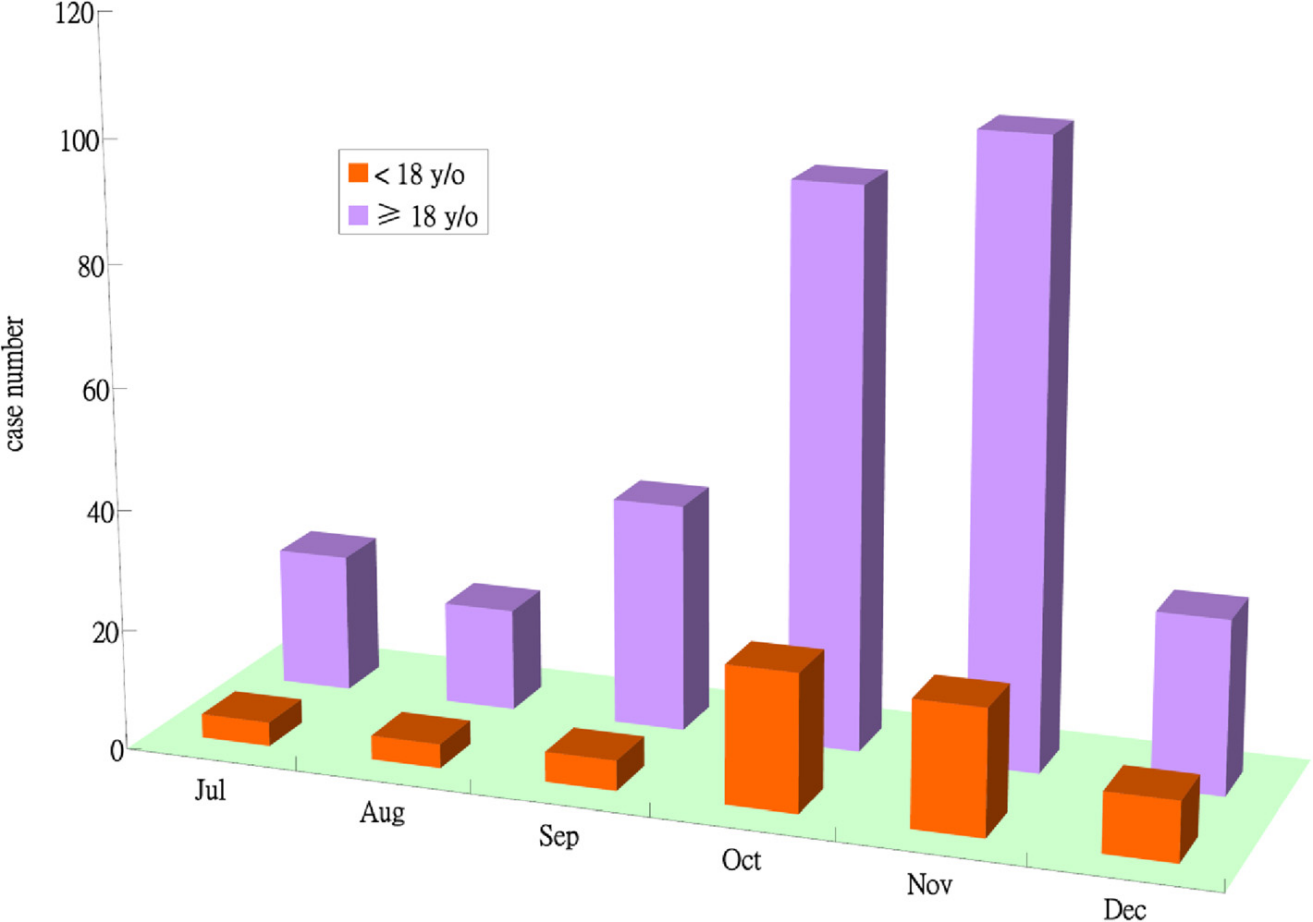
Monthly distributions of laboratory-confirmed dengue patients in southern Taiwan, 2007.

The peak period of this outbreak was in October-November (240, 63.8%) and there was no confirmed dengue case after Dec 31, 2007 (Figure 2). The enrolled suspected dengue cases during the study period consist of 100 children (≤ 18 years) and 481 adults. Among the 581 patients, 67 (67%) children and 309 (64.2%) adults were laboratory-positive. Patients who had laboratory indeterminate were excluded. The median age was 13 years in children and 48 years in adults. There were no gender predominance either in adult or pediatric groups (46.9% male in adult vs. 64.3% in children, p = 0.088).

The mean febrile duration in adults (5.1 ± 1.5 days) and children (6 ± 1.7 days) were similar (p = 0.459). Most of the patients visit our hospital within the 4th day of disease. The most common clinical presentations in either adult or pediatric dengue patients were myalgia (46.2% vs. 46.9%, p = 0.827), petechiae (49.2% vs. 34.3%, p = 0.275) and nausea/vomiting (44.7% vs. 31.1%. p = 0.144). However, almost half of the pediatric patients had itching skin, which is less frequent in adults (49.2% vs. 12.5%, p < 0.05). Gastrointestinal symptoms including nausea/vomiting and diarrhea were also leading complaints in this epidemic (Table 2).

**Table 2:** Clinical presentations and laboratory features of 376 dengue patients in first visit to clinic during the 2007 outbreak in southern Taiwan

	**≥18 y/o**	**<18 y/o**	P*
**Febrile days**	5.1 ± 1.5	6 ± 1.7	0.459
**Fever to clinic (days)**	4.1 ± 2.1	4 ± 1.7	0.522
**Symptoms/signs**			
**Myalgia**	46.9%	46.2%	0.827
**Petechiae**	34.3%	49.2%	0.275
**Headache**	31.1%	38.8%	0.116
**Itching skin**	12.5%	49.2%	<0.05
**Nausea/vomiting**	31.1%	44.7%	0.144
**Diarrhea**	23.4%	26.5%	0.707
**WBC (/cmm)**	3564 ± 1719	2971 ± 1761	0.006
**PLT (×10**^**3**^**/cmm)**	96 ± 51	102 ± 46	0.459
**AST (U/L)**	126 ± 132	166 ± 206	0.272
**ALT (U/L)**	81 ± 77	80 ± 101	0.492
**CRP (mg/L)**	20 ± 24	6 ± 11	0.011
**aPTT (sec)**	39 ± 6	44 ± 10	0.021

*Chi-square test or t-test, Figures were presented as mean ± standard deviation, WBC: white blood cell, PLT: platelet, AST: aspartate aminotransferase, ALT: alanine aminotransferase, CRP: C-reactive protein, aPTT: activated partial thrombin time.

The most notable laboratory findings included leukopenia, thrombocytopenia, prolonged aPTT, elevated serum levels of aminotransferase and low CRP. Lower WBC (2971 ± 1761/cmm vs. 3564 ± 1719/cmm, p = 0.006) and CRP level (6 ± 11 mg/L vs. 20 ± 24 mg/L, p = 0.011) and longer aPTT (39 ± 6 secs vs. 44 ± 10 secs, p = 0.021) were frequent to encounter in dengue-infected children (Table 2).

In comparison with laboratory-confirmed dengue-negative cases, dengue-positive patient had more headache (32.4% vs. 14%, p < 0.01), nausea/vomiting (33.5% vs. 15.8%, p < 0.01) and diarrhea (23.9% vs. 12.3%, p = 0.012). Dengue-positive cases also lower average WBC count (3458 vs. 5950/cmm, p < 0.01), platelet count (97 vs. 147 × 10^3^/cmm, p < 0.01), serum alanine aminotransferase (81 U/L vs. 134 U/L, p = 0.019) and CRP levels (18 vs. 36, p < 0.01). Moreover, the average aaPTT was longer in dengue-positive patients (40 secs vs. 31 secs, p = 0.003) (Table 3).

**Table 3:** Comparison of clinical and laboratory characteristics between dengue positive and negative cases during the 2007 outbreak in southern Taiwan

	**Dengue positive (n = 376)**	**Dengue negative (n = 114)**	P*
**Symptoms/signs**			
**Myalgia**	46.8%	40.4%	0.274
**Petechiae**	36.9%	29.8%	0.201
**Headache**	32.4%	14.0%	<0.01
**Itching skin**	8.6%	14.9%	0.075
**Nausea/vomiting**	33.5%	15.8%	<0.01
**Diarrhea**	23.9%	12.3%	0.012
**WBC (/cmm)**	3458 ± 3956	5950 ± 2851	<0.01
**PLT (K/cmm)**	97 ± 122	147 ± 85	<0.01
**AST (U/L)**	133 ± 293	111 ± 241	0.466
**ALT (U/L)**	81 ± 150	134 ± 341	0.019
**CRP (mg/L)**	18 ± 29	36 ± 60	<0.01
**aPTT (sec)**	40 ± 45	31 ± 3	0.003

*Chi-square test or t-test, Figures were presented as mean ± standard deviation, WBC: white blood cell, PLT: platelet, AST: aspartate aminotransferase, ALT: alanine aminotransferase, CRP: C-reactive protein, APTT: activated partial thrombin time.

To determine the predictive values of those common clinical and laboratory characteristics for laboratory-confirmed dengue infection, calculation were performed based on the clinical/laboratory features for predicting laboratory-confirmed dengue infection. The sensitivities of skin rash, myalgia, and positive tourniquet test are 59.2%, 46.8%, and 34.2%, while the specificities for above features are 75.4%, 53.5% and 100%, respectively. Rash and positive tourniquet test had high positive predictive value (88.8% and 100%, respectively) (Table 4). However, rashes appeared usually late (after febrile stage) in clinical course of dengue infection, and was not a good predictor of acute dengue infection. Besides, Tourniquet test was not routinely performed in clinical practice and the sensitivity was also too low for acute dengue infection. Therefore we measured several laboratory parameters as predictors of laboratory-confirmed dengue infections. No single laboratory test was good as enough in terms of positive predictive value. However, we found that the positive predictive value (PPV) for combination of leukopenia, thrombocytopenia, elevated aminotransferase and low CRP is 89.5% and negative predictive value (NPV) is 37.4%. Furthermore, the PPV of the combination was increased to 93.1% by adding prolonged aPTT. This combination served a good predictive marker for acute dengue infections (Table 5).

**Table 4:** Value of selected clinical features in predicting a laboratory-positive diagnosis of dengue in suspected dengue patients during the 2007 outbreak in southern Taiwan

**Laboratory feature**	**Dengue (+)**	**Dengue (−)**	**Sensitivity (%)**	**Specificity (%)**	**Positive predictive value (%)**	**Negative predictive value (%)**
(1) Fever	253/376	99/114	67.3	12.3	71.7	10.2
(2) Rash	222/376	28/114	59.2	75.4	88.8	35.8
(3) Petechiae	184/376	85/114	37	25.4	62.1	10.9
(4) Myalgia	268/376	53/114	46.8	53.5	76.9	23.4
(5) Nausea/vomiting	126/376	38/114	33.5	66.7	76.8	23.3
(6) Positive tourniquet test*	178/263	0/68	34.2	100	100	28.2

*Numbers were presented as positive cases/examined cases.

**Table 5:** Value of selected laboratory features in predicting a laboratory-positive diagnosis of dengue in suspected dengue patients during the 2007 outbreak in southern Taiwan

**Laboratory feature**	Dengue (+)*	Dengue (−)*	**Sensitivity (%)**	**Specificity (%)**	**Positive predictive value (%)**	**Negative predictive value (%)**
(1) WBC <4000/cmm	315/376	53/114	83.8	53.5	85.6	50.0
(2) PLT < 150 × 10^3^/cmm	314/376	52/114	83.5	54.4	85.8	50.0
(3) AST/ALT > 1.5	297/338	64/96	87.9	33.3	82.3	43.8
(4) CRP < 20 mg/L	335/357	75/102	93.8	26.5	81.7	55.1
(5) aPTT > 38 sec	227/301	37/80	75.4	53.8	86.0	36.8
(1) + (2)	276/376	52/114	73.4	45.6	84.1	34.2
(1) + (4)	291/357	34/102	81.5	66.7	89.5	50.7
(1) + (2) + (3)	228/338	38/96	67.5	60.4	85.7	34.5
(1) + (2) + (4)	255/357	27/102	71.4	73.5	90.4	42.4
(1) + (2) + (5)	209/301	26/80	69.4	67.5	88.9	37
(1) + (2) + (3) + (4)	221/338	26/96	65.4	72.9	89.5	37.4
(1) + (2) + (3) + (4) + (5)	172/301	11/80	49.5	86.3	93.1	31.2

WBC: white blood cell, PLT: platelet, AST: aspartate aminotransferase, ALT: alanine aminotransferase, CRP: C-reactive protein, aPTT: activated partial thrombin time. *Numbers were presented as positive cases / examined case.

## Discussion

For a dengue non-endemic area like Taiwan, early case identification is the key for effective dengue control. However, the revised 2009 symptom-based clinical management guideline from the World Health Organization did not require laboratory-confirmation. The revised classification of dengue cases is considered by many to be too broad [19,20]. Accurate diagnosis of dengue infection is of great important in non-endemic areas like Taiwan, since the goal of dengue control is not only to eliminate the occurrence of severe cases but also to confirm the possible dengue cases. However, the knowledge and practice of healthcare professionals in Taiwan seemed inadequate for the prompt case finding [21]. Universal laboratory for dengue infection are neither economic nor efficient for most countries. Therefore, there is a clinical need to determine who should take the diagnostic test, virological or serological, in daily clinical practice in Taiwan.

The early symptom/signs set of acute dengue virus infection is variable and it is difficult to distinguish it from other kinds of febrile illnesses [22,23]. Our study found that a combination of commonly routine blood tests including white blood count, platelet count, liver function tests, and CRP and coagulation profiles is useful in detecting laboratory-confirmed dengue infection. Previous study in Thailand showed that children with dengue were more likely to report anorexia, nausea, and vomiting and to have a positive tourniquet test. Dengue infected children had also lower total white blood cell counts, absolute neutrophil and absolute monocyte counts, and higher plasma AST and ALT levels [10]. A recent report also showed that patients with bleeding, decrease in total protein, increase in blood urea and decrease in lymphocyte proportion had higher odds for developing dengue hemorrhagic fever [24]. Simple clinical and laboratory markers can serve as an adjuvant in addition to history and physical examination, and also reduce the possible cost for universal laboratory diagnostic screening [14,24]. The symptoms combination identified here while having high positive predictive value still had low sensitivity. Therefore it may be a useful addition to the clinical evaluation and there is still a need to identify tests with better sensitivity and specificity. Besides, the accuracy of dengue diagnostic tests depends on the prevalence of dengue and time of sampling [25].

The difference in clinical manifestation between adult and pediatric dengue infections have been an important issue. A previous study in Taiwan showed that adult patients have higher incidences of arthralgia, myalgia, headache, abdominal pain and upper gastrointestinal bleeding. The adult dengue patient also had lower platelet counts, prothrombin time and serum albumin levels. The incidence of elevated alanine aminotransferase levels and prevalence of dengue hemorrhagic fever in adults are also higher than children [26]. The clinical manifestations in current study showed similarity between adult and pediatric patients during the outbreak. Whereas, the laboratory characteristics demonstrated significant differences of WBC (p = 0.006), CRP (p = 0.011) and aPTT (p = 0.021) between adult and pediatric dengue patients.

Dengue poses a substantial economic and disease burden in Southeast Asia [27]. The threshold for dengue admission in Taiwan is low, as showed in our study; nearly 80% of dengue patient were admitted to the hospital for 3–4 days. This finding is similar to Singapore [24], but much higher than other countries in the Americas and Asia. In a prospective health care facility-based study on disease burden and cost of dengue illnesses in eight endemic countries, around 45% were hospitalized for at least one day [28]. High admission rate for suspected dengue cases might compete the medical resources for other diseases and increase the burden of healthcare systems [20,27], simple predictive markers in this study could provide a screening tool in a non-endemic area of dengue. Dengue nonstructural protein 1 (NS1) antigen is an early antigen presenting in sera of Dengue patients and involves in the pathogenesis of dengue infection [29]. NS1 antigen strip has also been suggested as a rapid, easy-to-perform, sensitive, and specific test for the early diagnosis of dengue infection after the onset of fever [30]. Our predictive system could be either used to decide the priority of NS1 antigen strip usage or to increase the sensitivity/ specificity of the test.

The overall disease severity in this outbreak is relatively lower than other studies. One of the possible explanations is that most of our patients were infected by dengue virus serotype 1. Dengue virus serotype 2 has been associated with risk factors of developing dengue hemorrhagic fever/dengue shock syndrome [29,31]. Besides, high accessibility to medical services and low admission threshold provide early intervention and prevent further morbidity/mortality. This practice is in line with the 2009 WHO clinical management guideline, which also encourage admitting patients with danger signs to the medical facilities [20].

There are several limitations in our study. Firstly, the retrospective and hospital-based design might not reflect the whole dengue-infected groups. However, these results provide useful references for clinician practicing in a non-endemic area of dengue. Secondly, the symptoms combination identified while having high positive predictive value still had low sensitivity. Therefore, while it may be a useful addition to the clinical evaluation, there is still a need to identify tests with better sensitivity and specificity. Finally, the study was conducted in a population with high prevalence of dengue infection and therefore the high positive predictive value will not be replicated in a low prevalence population. The accuracy of these predictive markers might be subject to change in accordance to the prevalence of dengue infection and alertness of healthcare professionals. There is still no single clinical and laboratory marker available for predicting dengue infection, even the new WHO guideline [32].

## Conclusions

Early diagnosis of dengue infection remains a challenge to clinicians around the world. The positive predictive value for laboratory-confirmed dengue infection with combination of leukopenia (< 4000/cmm), thrombocytopenia (< 150 × 103/cmm), prolonged aPTT (> 38 sec), elevated aminotransferase (AST/ALT > 1.5) and low CRP (< 20 mg/L) is 93.1%. These clinical and laboratory findings may serve as predictive markers to promote early diagnosis of dengue infection in Taiwan.

## Competing interests

None of the authors have professional, personal, or financial conflicts of interest to report.

## Authors’ contributions

TSH, SMW collected information, designed and organized the structure of the contents and wrote the manuscript. YSL, CCL reviewed literature, discussed and suggested the contents as well as edited the manuscript. All the authors read and approved the final manuscript.
